# Supplementary material for: Reducing blood product wastage: Insights from a retrospective study in a tertiary health facility
Source: PLoS One. 2026 Mar 25;21(3):e0344521. doi: 10.1371/journal.pone.0344521 (PMC13016284; doi:10.1371/journal.pone.0344521)
Supplement: S1 Table — (DOCX) [file pone.0344521.s001.docx]

**Supplementary S1 Table. Summary of data collected from ninety-eight departments aggregated into nine broader categories.**

| Main departments | Sub-departments | RBCs | | Platelets | | FFP | | Cryoprecipitate | | Total | |
| --- | --- | --- | --- | --- | --- | --- | --- | --- | --- | --- | --- |
|  |  | **n** | **n%** | **n** | **n%** | **n** | **n%** | **n** | **n%** | **n** | **n%** |
| Adult CVOR | NA | 112 | 0.28 | 56 | 0.31 | 144 | 0.90 | 804 | 13.10 | 1116 | 1.39 |
| AED | NA | 4380 | 10.93 | 636 | 3.51 | 1052 | 6.56 | 353 | 5.75 | 6421 | 7.99 |
| Children's Specialist Hospital wards and departments | CSH 2-1 | 1.21 | 190 | 1.05 | 173 | 1.08 | 276 | 4.5 | 1125 | 1.4 | 486 |
|  | CSH 2-2 | 0.61 | 37 | 0.2 | 36 | 0.22 | 8 | 0.13 | 326 | 0.41 | 245 |
|  | CSH 3-1 | 0.6 | 106 | 0.58 | 42 | 0.26 | 9 | 0.15 | 398 | 0.5 | 241 |
|  | CSH 3-2 | 0.41 | 146 | 0.81 | 39 | 0.24 | 9 | 0.15 | 358 | 0.45 | 164 |
|  | CSH 3-3 | 2.51 | 1540 | 8.49 | 162 | 1.01 | 22 | 0.36 | 2732 | 3.4 | 1008 |
|  | CSH 3-4 | 1.1 | 523 | 2.88 | 59 | 0.37 | 4 | 0.07 | 1027 | 1.28 | 441 |
|  | CSH 4-2 | 0.35 | 82 | 0.45 | 52 | 0.32 | 3 | 0.05 | 277 | 0.34 | 140 |
|  | CSH 4-3 | 0.53 | 24 | 0.13 | 39 | 0.24 | 52 | 0.85 | 329 | 0.41 | 214 |
|  | CSH 4-4 | 0.61 | 129 | 0.71 | 88 | 0.55 | 29 | 0.47 | 489 | 0.61 | 243 |
|  | CSH ED | 0.01 | 0 | 0 | 1 | 0.01 | 0 | 0 | 4 | 0 | 3 |
|  | CSH Hybrid | 0.04 | 4 | 0.02 | 1 | 0.01 | 1 | 0.02 | 21 | 0.03 | 15 |
|  | CSH PCIU | 0 | 0 | 0 | 0 | 0 | 0 | 0 | 2 | 0 | 2 |
|  | CSH PCVICU | 2.53 | 536 | 2.96 | 310 | 1.93 | 223 | 3.63 | 2082 | 2.59 | 1013 |
|  | CSH PICU | 0.48 | 146 | 0.81 | 74 | 0.46 | 16 | 0.26 | 429 | 0.53 | 193 |
| OR | MAIN OR | 375 | 0.94 | 36 | 0.2 | 206 | 1.29 | 49 | 0.8 | 666 | 0.83 |
|  | ICU Pharm | 0 | 0 | 0 | 0 | 0 | 0 | 8 | 0.13 | 8 | 0.01 |
|  | Minor OR | 0 | 0 | 1 | 0.01 | 0 | 0 | 0 | 0 | 1 | 0 |
|  |  |  |  |  |  |  |  |  |  |  |  |
| Main Hospital wards | MH 2-1 | 362 | 0.9 | 68 | 0.38 | 158 | 0.99 | 57 | 0.93 | 645 | 0.8 |
|  | MH 2-2 | 430 | 1.07 | 77 | 0.42 | 208 | 1.3 | 91 | 1.48 | 806 | 1 |
|  | MH 2-3&4 | 675 | 1.68 | 91 | 0.5 | 227 | 1.42 | 18 | 0.29 | 1011 | 1.26 |
|  | MH 2-5 | 355 | 0.89 | 66 | 0.36 | 210 | 1.31 | 116 | 1.89 | 747 | 0.93 |
|  | MH 2-6 | 324 | 0.81 | 64 | 0.35 | 138 | 0.86 | 33 | 0.54 | 559 | 0.7 |
|  | MH 3-1 A | 15 | 0.04 | 4 | 0.02 | 4 | 0.02 | 6 | 0.1 | 29 | 0.04 |
|  | MH 3-1 P | 0 | 0 | 1 | 0.01 | 0 | 0 | 0 | 0 | 1 | 0 |
|  | MH 3-1A | 694 | 1.73 | 1128 | 6.22 | 98 | 0.61 | 41 | 0.67 | 1961 | 2.44 |
|  | MH 3-1B | 752 | 1.88 | 946 | 5.22 | 154 | 0.96 | 112 | 1.82 | 1964 | 2.44 |
|  | MH 3-2 | 1 | 0 | 1 | 0.01 | 0 | 0 | 0 | 0 | 2 | 0 |
|  | MH 3-2A | 573 | 1.43 | 949 | 5.23 | 49 | 0.31 | 43 | 0.7 | 1614 | 2.01 |
|  | MH 3-2B | 307 | 0.77 | 130 | 0.72 | 159 | 0.99 | 1 | 0.02 | 597 | 0.74 |
|  | MH 3-3 | 738 | 1.84 | 480 | 2.65 | 137 | 0.85 | 76 | 1.24 | 1431 | 1.78 |
|  | MH 3-4 | 1108 | 2.76 | 1142 | 6.3 | 145 | 0.9 | 145 | 2.36 | 2540 | 3.16 |
|  | MH 3-5 | 612 | 1.53 | 67 | 0.37 | 219 | 1.37 | 18 | 0.29 | 916 | 1.14 |
|  | MH 3-6 | 409 | 1.02 | 33 | 0.18 | 253 | 1.58 | 34 | 0.55 | 729 | 0.91 |
|  | MH 3-7 | 352 | 0.88 | 67 | 0.37 | 104 | 0.65 | 18 | 0.29 | 541 | 0.67 |
|  | MH 3-8 | 385 | 0.96 | 41 | 0.23 | 142 | 0.89 | 10 | 0.16 | 578 | 0.72 |
|  | MH 4-1 | 54 | 0.13 | 1 | 0.01 | 0 | 0 | 0 | 0 | 55 | 0.07 |
|  | MH 4-1 HDU | 101 | 0.25 | 6 | 0.03 | 14 | 0.09 | 14 | 0.23 | 135 | 0.17 |
|  | MH 4-3 | 224 | 0.56 | 16 | 0.09 | 52 | 0.32 | 0 | 0 | 292 | 0.36 |
|  | MH 4-4 | 131 | 0.33 | 22 | 0.12 | 12 | 0.07 | 34 | 0.55 | 199 | 0.25 |
|  | MH 4-5 | 466 | 1.16 | 32 | 0.18 | 56 | 0.35 | 10 | 0.16 | 564 | 0.7 |
|  | MH 4-6 EDU | 99 | 0.25 | 14 | 0.08 | 54 | 0.34 | 0 | 0 | 167 | 0.21 |
|  | ADULT BRON | 13 | 0.03 | 4 | 0.02 | 0 | 0 | 1 | 0.02 | 18 | 0.02 |
|  | Adult OTU | 59 | 0.15 | 17 | 0.09 | 4 | 0.02 | 0 | 0 | 80 | 0.1 |
|  | BB | 66 | 0.16 | 8 | 0.04 | 25 | 0.16 | 29 | 0.47 | 128 | 0.16 |
|  | MH A-CVICU | 1082 | 2.7 | 271 | 1.49 | 846 | 5.28 | 440 | 7.17 | 2639 | 3.28 |
|  | MH CATH EP | 27 | 0.07 | 2 | 0.01 | 18 | 0.11 | 12 | 0.2 | 59 | 0.07 |
|  | MH CCU | 608 | 1.52 | 141 | 0.78 | 451 | 2.81 | 110 | 1.79 | 1310 | 1.63 |
|  | MH CVSSD | 157 | 0.39 | 11 | 0.06 | 69 | 0.43 | 25 | 0.41 | 262 | 0.33 |
|  | MH DCU | 801 | 2 | 140 | 0.77 | 156 | 0.97 | 4 | 0.07 | 1101 | 1.37 |
|  | MH Dial | 34 | 0.08 | 0 | 0 | 0 | 0 | 0 | 0 | 34 | 0.04 |
|  | MH ICU | 5389 | 13.44 | 2340 | 12.9 | 5098 | 31.81 | 1651 | 26.89 | 14478 | 18.01 |
|  | MH NCCU | 895 | 2.23 | 942 | 5.19 | 433 | 2.7 | 116 | 1.89 | 2386 | 2.97 |
|  | MH NCSD | 74 | 0.18 | 14 | 0.08 | 33 | 0.21 | 12 | 0.2 | 133 | 0.17 |
|  | MH PED DCU | 298 | 0.74 | 42 | 0.23 | 116 | 0.72 | 0 | 0 | 456 | 0.57 |
|  | MH Ped OTU | 167 | 0.42 | 181 | 1 | 3 | 0.02 | 0 | 0 | 351 | 0.44 |
|  | MH SU | 103 | 0.26 | 1 | 0.01 | 2 | 0.01 | 26 | 0.42 | 132 | 0.16 |
|  | Hem BMT | 2 | 0 | 1 | 0.01 | 0 | 0 | 0 | 0 | 3 | 0 |
|  | Hema BMT | 1 | 0 | 0 | 0 | 0 | 0 | 0 | 0 | 1 | 0 |
|  | EMH ICU | 4 | 0.01 | 1 | 0.01 | 6 | 0.04 | 0 | 0 | 11 | 0.01 |
|  | EMH BMT | 2 | 0 | 3 | 0.02 | 0 | 0 | 0 | 0 | 5 | 0.01 |
|  | MH ER CT | 0 | 0 | 1 | 0.01 | 0 | 0 | 0 | 0 | 1 | 0 |
| NULL |  | 3323 | 8.29 | 1920 | 10.59 | 1110 | 6.93 | 509 | 8.29 | 6862 | 8.54 |
| Pediatrics department | OB Triage | 467 | 1.16 | 14 | 0.08 | 104 | 0.65 | 13 | 0.21 | 598 | 0.74 |
|  | PCVOR | 21 | 0.05 | 15 | 0.08 | 19 | 0.12 | 101 | 1.65 | 156 | 0.19 |
|  | PCVOR DCU | 1 | 0 | 0 | 0 | 0 | 0 | 0 | 0 | 1 | 0 |
|  | Ped Dialys | 1 | 0 | 0 | 0 | 0 | 0 | 0 | 0 | 1 | 0 |
|  | PED ED | 886 | 2.21 | 539 | 2.97 | 134 | 0.84 | 23 | 0.37 | 1582 | 1.97 |
|  | PED ONC/HE | 1 | 0 | 0 | 0 | 0 | 0 | 0 | 0 | 1 | 0 |
|  | PED PULM | 158 | 0.39 | 76 | 0.42 | 20 | 0.12 | 1 | 0.02 | 255 | 0.32 |
|  | Ped Renal | 14 | 0.03 | 0 | 0 | 0 | 0 | 0 | 0 | 14 | 0.02 |
|  | Peds OR | 10 | 0.02 | 2 | 0.01 | 6 | 0.04 | 0 | 0 | 18 | 0.02 |
|  | OPD PED | 0 | 0 | 1 | 0.01 | 0 | 0 | 0 | 0 | 1 | 0 |
| Rehabilitation Wards | RH 1-1 | 123 | 0.31 | 15 | 0.08 | 31 | 0.19 | 0 | 0 | 169 | 0.21 |
|  | RH 1-2 | 121 | 0.3 | 23 | 0.13 | 29 | 0.18 | 6 | 0.1 | 179 | 0.22 |
|  | RH 1-3 | 76 | 0.19 | 76 | 0.42 | 4 | 0.02 | 16 | 0.26 | 172 | 0.21 |
|  | RH 2-1 | 138 | 0.34 | 109 | 0.6 | 28 | 0.17 | 4 | 0.07 | 279 | 0.35 |
|  | RH 2-2 | 300 | 0.75 | 32 | 0.18 | 161 | 1 | 16 | 0.26 | 509 | 0.63 |
|  | RH 2-3 | 52 | 0.13 | 40 | 0.22 | 2 | 0.01 | 0 | 0 | 94 | 0.12 |
|  | RH 3-1 | 49 | 0.12 | 32 | 0.18 | 9 | 0.06 | 0 | 0 | 90 | 0.11 |
| Women Specialist Hospital wards | WSH 1-H | 338 | 0.84 | 162 | 0.89 | 59 | 0.37 | 9 | 0.15 | 568 | 0.71 |
|  | WSH 2-1 | 902 | 2.25 | 53 | 0.29 | 330 | 2.06 | 64 | 1.04 | 1349 | 1.68 |
|  | WSH 2-2 | 741 | 1.85 | 30 | 0.17 | 152 | 0.95 | 13 | 0.21 | 936 | 1.16 |
|  | WSH 2-3 | 479 | 1.19 | 187 | 1.03 | 131 | 0.82 | 20 | 0.33 | 817 | 1.02 |
|  | WSH 2-4 | 429 | 1.07 | 147 | 0.81 | 99 | 0.62 | 7 | 0.11 | 682 | 0.85 |
|  | WSH 2-N1 | 0 | 0.00 | 0 | 0.00 | 1 | 0.01 | 0 | 0.00 | 1 | 0.00 |
|  | WSH 3-1 | 573 | 1.43 | 46 | 0.25 | 222 | 1.39 | 26 | 0.42 | 867 | 1.08 |
|  | WSH 3-3 | 670 | 1.67 | 14 | 0.08 | 59 | 0.37 | 0 | 0 | 743 | 0.92 |
|  | WSH 3-4 | 257 | 0.64 | 24 | 0.13 | 71 | 0.44 | 4 | 0.07 | 356 | 0.44 |
|  | WSH ICU D | 223 | 0.56 | 61 | 0.34 | 276 | 1.72 | 49 | 0.8 | 609 | 0.76 |
|  | WSH NICU 2 | 108 | 0.27 | 14 | 0.08 | 15 | 0.09 | 0 | 0 | 137 | 0.17 |
|  | WSH NICU 3 | 944 | 2.35 | 705 | 3.89 | 520 | 3.24 | 20 | 0.33 | 2189 | 2.72 |
|  | WSH OR | 72 | 0.18 | 8 | 0.04 | 37 | 0.23 | 27 | 0.44 | 144 | 0.18 |
|  | KFMC L&D | 381 | 0.95 | 29 | 0.16 | 62 | 0.39 | 42 | 0.68 | 514 | 0.64 |
|  | KFMC FM | 2 | 0 | 0 | 0 | 0 | 0 | 0 | 0 | 2 | 0 |
|  | WSH DCU | 0 | 0 | 0 | 0 | 1 | 0.01 | 0 | 0 | 1 | 0 |
|  | OPD Gyn | 0 | 0 | 1 | 0.01 | 6 | 0.04 | 0 | 0 | 7 | 0.01 |
